# Sensing Circuit Design Techniques for RRAM in Advanced CMOS Technology Nodes

**DOI:** 10.3390/mi12080913

**Published:** 2021-07-30

**Authors:** Donglin Zhang, Bo Peng, Yulin Zhao, Zhongze Han, Qiao Hu, Xuanzhi Liu, Yongkang Han, Honghu Yang, Jinhui Cheng, Qingting Ding, Haijun Jiang, Jianguo Yang, Hangbing Lv

**Affiliations:** 1 Key Laboratory of Microelectronic Devices Integrated Technology, Institute of Microelectronics of Chinese Academy of Sciences, Beijing 100029, China; zhangdonglin20@mails.ucas.ac.cn (D.Z.); zhaoyulin@ime.ac.cn (Y.Z.); hanzhongze20@mails.ucas.ac.cn (Z.H.); qhu@mail.ustc.edu.cn (Q.H.); xuanzhi@mail.ustc.edu.cn (X.L.); hommyoun@163.com (H.Y.); chengjh0903@foxmail.com (J.C.); dingqingting@ime.ac.cn (Q.D.); lvhangbing@ime.ac.cn (H.L.); 2 School of Microelectronics, University of Chinese Academy of Sciences, Beijing 100049, China; 3 Zhejiang Lab, Hangzhou 311121, China; pengb806@nenu.edu.cn (B.P.); hanyk@zhejianglab.com (Y.H.); jianghaijun@zhejianglab.com (H.J.); 4 School of Microelectronics, University of Science and Technology of China, Hefei 230026, China

**Keywords:** RRAM, reference schemes, sensing schemes, BL-enhancing schemes

## Abstract

Resistive random access memory (RRAM) is one of the most promising new nonvolatile memories because of its excellent properties. Moreover, due to fast read speed and low work voltage, it is suitable for seldom-write frequent-read applications. However, as technology nodes shrink, RRAM faces many issues, which can significantly degrade RRAM performance. Therefore, it is necessary to optimize the sensing schemes to improve the application range of RRAM. In this paper, the issues faced by RRAM in advanced technology nodes are summarized. Then, the advantages and weaknesses in the novel design and optimization methodologies of sensing schemes are introduced in detail from three aspects, the reference schemes, sensing amplifier schemes, and bit line (BL)-enhancing schemes, according to the development of technology in especially recent years, which can be the reference for designing the sensing schemes. Moreover, the waveforms and results of each method are illustrated to make the design easy to understand. With the development of technology, the sensing schemes of RRAM become higher speed and resolution, low power consumption, and are applied at advanced technology nodes and low working voltage. Now, the most advanced nodes the RRAM applied is 14 nm node, the lowest working voltage can reach 0.32 V, and the shortest access time can be only a few nanoseconds.

## 1. Introduction

In recent years, Moore’s Law has been pushed down to three nanometers, or even one nanometer. Nevertheless, the technology nodes of non-volatile memory lag far behind. At present, the mainstream nonvolatile memory is Flash [1,2,3,4]. It is low cost and easy to achieve large capacity. However, after the 40 nm technology node, the flash memory cell is prone to a large amount of leakage and poor performance, because of its device characteristics based on floating gate storage.

In order to overcome the traditional Flash scaling issue, there are two main technical solutions: one is based on the traditional flash, through further reducing the cell size to improve the storage density; the other one is to introduce emerging memory technology, such as magnetoresistive random access memory (MRAM) [5,6,7,8,9], ferroelectric random access memory (FRAM) [10], phase-change memory (PCM) [11,12], and RRAM [13,14,15], etc.

FRRAM has become the most promising emerging nonvolatile memory, due to its simple structure, CMOS compatibility, good scalability [16,17,18], fast operation speed [19,20,21], low operating voltage, small operating current [22,23,24,25,26,27,28,29,30], and high reliability [31,32,33].

The concept of RRAM was first proposed by Professor Shaotang Tsai in 1971 [34], but it was not until 2008 that HP Labs presented the first memristor element [35]. Afterward, memristor has become a hot research topic. More than 100 research institutions and companies have carried out RRAM research, and a large number of relevant literature is published every year. RRAM has been used in some products. Panasonic realized an embedded application of RRAM on MCU in 2013. Crossbar claims that they have successfully developed some RRAM IPs. Recently, it has been reported that RRAM in advanced technology nodes (below 28 nm) is close to mass production. Moreover, the storage capacity of RRAM has been 16 GB [36,37,38], or even 32 GB [39], which indicates that RRAM can also be used as stand-alone storage.

Despite the good progress in RRAM research, as RRAM wants to replace Flash and, essentially, mass production, there are still many issues to be solved.

RRAM exhibits different resistance states depending on the voltage, which is applied to the cell. According to the different resistance values, it is divided into high resistance state (HRS)and low resistance state (LRS) to store ’0’ and ’1’. In essence, it depends on the formation and dissolution of the conductive filaments inside RRAM. However, the formation of conductive filaments is not easy to control, which is closely related to the voltage/current applied to the cell. Therefore, the resistance distribution of RRAM may have significant variation. Moreover, when reading the RRAM cell, it may cause its resistance to be changed or deviated from the stored value. The resistance of RRAM is sensitive to temperature, which makes it difficult to read with high reliability. Beyond that, it may have a sneak current in the RRAM array, which will increase the power consumption of the scheme, and even causes misreading. In addition, RRAM peripheral schemes are also facing lots of challenges at advanced technology nodes. As the technology shrinks, the working voltage decreases. Circuit design becomes more difficult. Moreover, the process variations become serious and it may lead to a more considerable mismatch and offset current in RRAM sensing schemes.

Consequently, the peripheral schemes should be designed carefully and optimized fully to improve the performance of RRAM. The peripheral schemes of RRAM consist of three main parts: cell and array schemes, sensing schemes, and writing schemes. The performances of cell and array schemes are limited by the cell characters mainly. Moreover, a large proportion of writing schemes adopt write-verify-write mode, which is heavily dependent on the sensing schemes. Therefore, the sensing schemes are of great significance in the design of the RRAM chip, which greatly affects its performance.

In recent years, many reviews on RRAM have been conducted. In 2017, Huaqiang Wu et al., produced a highly systematic review on RRAM [40]. There are also some reviews on RRAM-resistive switching behavior, mechanism, and materials [41,42,43,44]. However, reviews dedicated to RRAM peripheral schemes, especially for reading schemes, are limited. Therefore, writing a paper, which introduces the peripheral schemes in the last ten years, especially for the sensing schemes, is necessary. This paper presents the sensing schemes in three parts—reference schemes, sensing amplifier schemes, and the BL-enhancing schemes. First, a variety of reference schemes are introduced, ranging from the simplest parallel type to the hybrid type and multiple configurable dummy cells. Then, this paper presents some novel technologies of sensing amplifier schemes on reducing power consumption, enlarging sensing margin, improving voltage resolution, ultra-low working voltage, etc. Finally, some methods on BL-enhancing schemes are introduced, including process temperature detecting, BL precharge speed enhancement, and lowing the ripple on the BL.

In Section 2, this part will introduce the current design challenges of RRAM sensing schemes in advanced technology nodes in detail and show the collaborative optimization of schemes and technologies in the last 10 years. In Section 3, the design and optimization of the sensing schemes of RRAM will be introduced in detail. In Section 4, this part will look into the future of RRAM sensing schemes design.

## 2. The Design Challenges of RRAM

The sensing schemes include three parts mainly. They are reference schemes, sensing amplifier schemes, and BL-enhancing schemes. Reference schemes provide an accurate reference voltage/current during the sensing period. Sensing amplifier schemes mainly sample and amplify the cell current/voltage to output a digital value. BL-enhancing schemes are used to stabilize BL voltage and speed up the charging of BL.

This part will introduce the design challenges in RRAM and their impact on sensing schemes.

### 2.1. The Working Voltage Decreases as Technology Shrink

**Figure 1:**
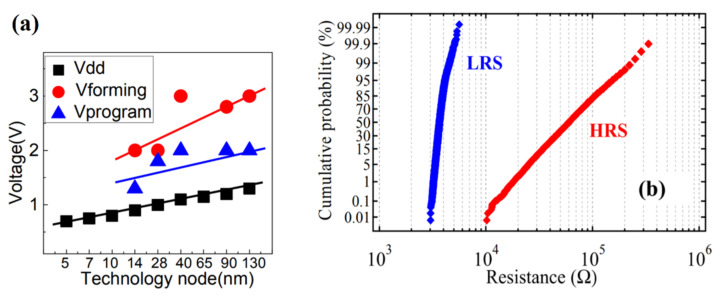
(**a**) The operation voltage of core device and RRAM cell at different technology nodes [45]. (**b**) The resistance distributions [48].

With the technology node shrinking, the supply voltage is getting lower and lower. As shown in Figure 1a, for the LVT of the 28 nm node, the supply voltage (VDD) is already less than 1 V. However, the operating voltage of the RRAM cell is much higher than the supply voltage, especially for the forming voltage. The voltage reduction will also cause the degradation of the sensing window and decrease the voltage headroom of sensing schemes, which further increases the complexity of the sensing schemes design [45,46,47].

### 2.2. Sneak Current Issues

**Figure 2:**
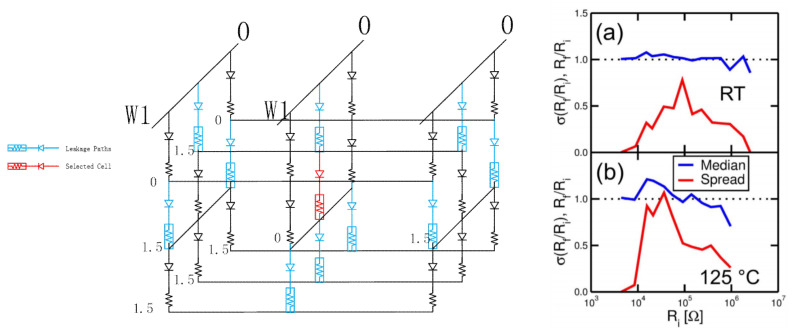
(**a**) Sneak current issues of cross-point RRAM [49]. (**b**) The average value and standard deviation of Rf (final resistance measured at RT)/Ri (initial resistance measured at RT) across various Ri [53].

In order to improve the integration density of RRAM, a memory device and an access device are stacked to form a three-dimensional cross-point structure. When the selected cell is read in the three-dimensional cross-point structure, the read current on the selected BL will be disturbed by the leakage current on the unselected cell, making it difficult to distinguish the reading signal accurately. Figure 2a shows the sneak current issue of the cross-point RRAM [12,49,50,51,52].

### 2.3. IR Drop Issues

As the capacity and density of the RRAM array increase, the parasitic resistance and capacitance on the data path of a cell will increase, which causes an apparent voltage or current drop. The effective voltage or current applied to the cell is significantly reduced, which will decrease the sensing window. Using BL-enhancing schemes can compensate for certain voltage drops [54,55,56,57,58,59].

### 2.4. Resistance Variability Issues

In the operation process of Forming, Set, and Reset, the formation and dissolution of conductive filament in the RRAM cell are random. The random process introduces variability into the switching characteristics of the RRAM. It is also found that the uniformity of conductive filament formation in the RRAM cell is reduced because the overshoot current on the long BL is unable to be controlled. These are the reasons for the wide resistance distribution of RRAM. The wide range of HRS (high resistance state) and LRS (low resistance state) resistance values poses a challenge to high-speed reading operations. Because of the variability, the RH (the resistance of the cell state is HRS)/RL (the resistance of the cell state is LRS; R-ratio) becomes small, making the sensing window small and the reference schemes hard to design. Figure 1b shows that the distributions of LRS and HRS [48,60,61,62,63].

### 2.5. Temperature Dependence of RRAM Cell

At high temperatures, both the high resistance and low resistance of RRAM will drift towards low resistance. The high resistance state will drift more severely if the reference is fixed or cannot adapt to temperature changes well, which may cause the misreading of RRAM. Therefore, the reference of the sensing amplifier must consider the temperature effect of the RRAM to ensure that the reference voltage is in the middle of HRS and LRS. Figure 2b shows that the maximum drift of Rf/Ri is 1.07 and 1.21 at 25 °C (RT) and 125 °C, respectively, which makes the design of sensing schemes more difficult [53,64,65,66,67].

### 2.6. Read Disturb

The read operation is carried out by applying a certain read current or read voltage to the RRAM, which may cause the resistance to deviate from the original value and even change the RRAM data. Therefore, the reading operation should be as quick as possible. Moreover, the voltage applied to the cell should be as small as possible to avoid reading disturb issues [49]. It presents a significant challenge on BL-enhancing schemes and sensing schemes [68,69,70,71].

### 2.7. Sensing Margin Degradation

With technology scaling, the process variations increase. VDD and the read cell current (Icell) decrease. These factors all lead to the continuous decline of RRAM’s sensing margin [72]. Maintaining a target sensing margin is a challenge, especially at advanced technology nodes [73].

### 2.8. The Offset Current Increases in the Sense Circuit

Offset current is caused by many factors, including parasitic capacitors and resistors on the data path, the mismatch between transistors, and the offset voltage of the sensing schemes. It may decrease the sensing window, and the reading speed will be seriously affected.

It is an important method to optimize the performance of RRAM by circuit and technology cooperation. Recently, there is much literature on optimizing RRAM by circuit design. Table 1 is a summary of articles related to RRAM peripheral schemes in the past ten years. It shows that peripheral schemes are continuously optimized and iterated with technology nodes [74,75].

## 3. Sensing Circuit Design Techniques for RRAM

### 3.1. Reference Schemes

An accurate reference current is critical to the performance of the sensing schemes. If the reference voltage or current has some slight deviations, it may affect the sensing margin and operation speed. If the deviation is significant, it may even cause misreading.

Besides, the resistance of the RRAM cell will change with temperature and process variations. To obtain a larger sensing margin, the RRAM reference schemes should track the variations in temperature and process. The RRAM cell can be used to generate the reference signal, which can resist the variations of the process, temperature, and voltage.

**Figure 3:**
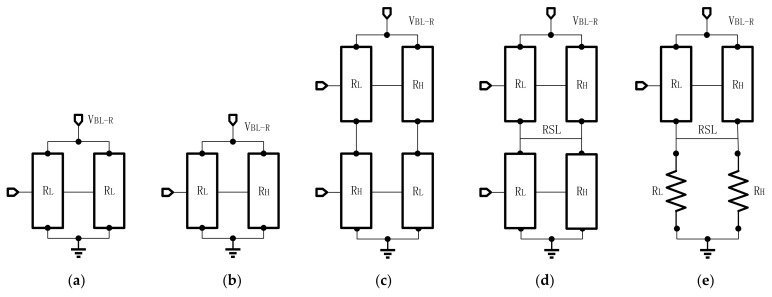
Different connection modes: (**a**) parallel; (**b**) R_H_ + R_L_ Avg; (**c**) series-parallel; (**d**) parallel-series; (**e**) hybrid reference.

For traditional reference cells, there are three connection methods that are widely used as shown in Figure 3. The most common methods are the parallel and RH + RL average methods. However, if the resistance of one of the reference cells offsets the targe value too much, it may lower the equivalent impedance of the entire reference circuit. Therefore, series-parallel is used to relieve this disturb. However, its output current value is $\frac{2V}{\left(R_{H}+R_{L}\right)}$, which is different from $\frac{1}{2}(\frac{V}{R_{H}}+\frac{V}{R_{L}})$. As the R-ratio increases, the reference will have a more significant deviation from the mean reference current. Therefore, Meng-Fan Chang proposed a parallel-series reference cell (PSRC), which is shown in Figure 3d [76]. Its output current value is $\frac{1}{2}(\frac{V}{R_{H}}+\frac{V}{R_{L}})$. Moreover, the PSRC achieved 29–32% and 27–56% smaller σ/μ values than conventional schemes.

Nevertheless, the tighter reference current generated by the reference circuit is wanted. Because the reference current of the linear resistor is the tightest, using some linear resistors to replace RRAM dummy cells, some temperature and process tracking ability is sacrificed to achieve better linearity. Therefore, Qiao wang et al., proposed a hybrid read reference structure (HRRS) [77]. Two linear resistors are used instead of the resistors in PSRC shown in Figure 3e.

**Figure 4:**
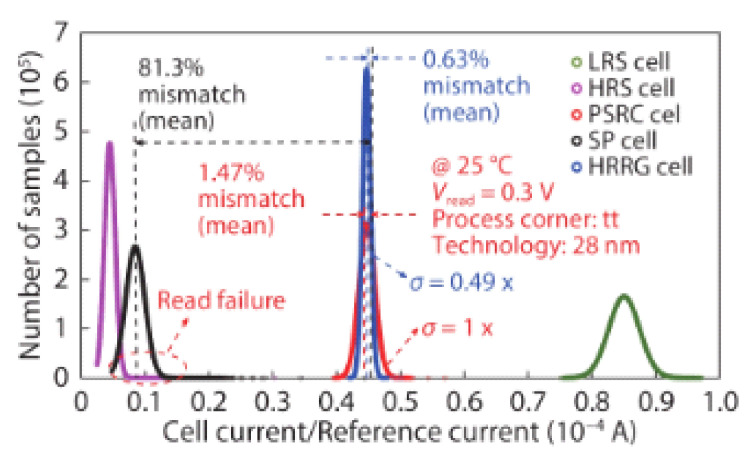
The distributions of cell current and the reference current [77].

Figure 4 shows the reference current generated by the SP, PSRC, and HRRG schemes. The ideal current should be in the middle of HRS and LRS, but the SP reference scheme is shifted to the state of HRS and causes overlap, which may cause reading errors. PSRC and HRRS results are basically the same. However, HRRS reduces the offset by 49% relative to PSRC, which shows that the current distribution is tight. Therefore, by using the HRRS reference scheme, the sensing margin will be enlarged, and the read operation’s accuracy will be improved.

**Figure 5:**
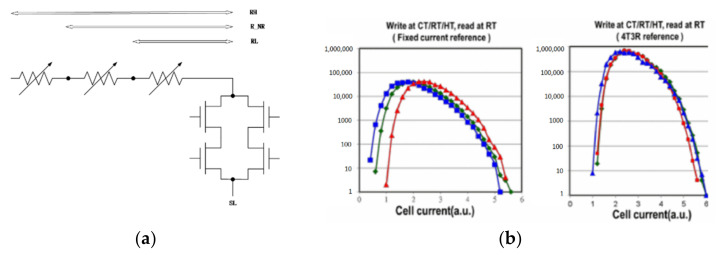
(**a**) 4T3R scheme; (**b**) The cell current distribution, the number of cell at different cell current [78].

In addition to using traditional liner resistance, Chung-Cheng Chou et al., proposed a self-tracking 4T3R reference scheme with thin film resistors shown in Figure 5a [78]. The two shunted NMOS pairs were connected in series to reflect the temperature effect, using 3R to emulate three states: the minimum HRS, the maximum LRS, and the level for a normal read. A temperature-invariant current flows through the scheme to adjust the reference current level if thin film resistors deviate from the target. Figure 5b shows the Icell read at RT (25 °C) after written at HT (120 °C)/RT/CT (−40 °C), respectively.

This reference scheme needs an extra temperature invariant current to adjust the reference current level if the thin film resistance deviates from the target, which increases the complexity of the scheme design.

**Figure 6:**
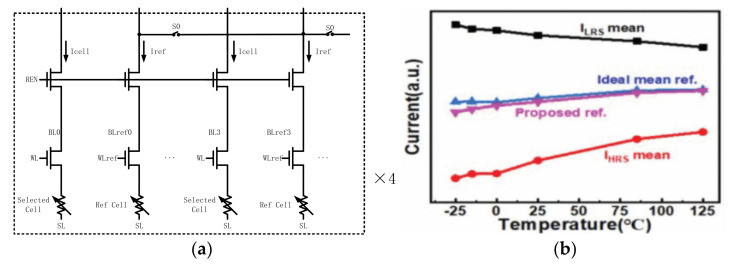
(**a**) The multiple dummy cells; (**b**) the read reference under different temperatures [79].

However, the previous reference schemes only use a few cells to generate the reference signal. If a cell fails, it will cause a large number of reading errors, because these reference cells are shared by other sensing schemes. In order to solve the tail-bit issues in the reference circuit, Jianguo Yang et al., proposed multiple configurable dummy cell reference generator schemes (MCDC), as shown in Figure 6 [79].

It uses multiple dummy 1T1R cells and ensures that each one is configurable. It uses 16 dummy cells in parallel as the reference when S0 is on, shown in Figure 6a. If a tail-bit cell appears, it can be configured to have more HRS cells or LRS cells to correct this error. Therefore, the problem of collapse can be solved. Moreover, the reference current tracks the temperature variations well. Figure 6b shows that the current generated by the reference circuit is extremely close to the ideal value. However, the operation of this scheme is a little bit complex and asks for a high requirement for the peripheral scheme.

### 3.2. Sensing Amplifier Schemes

RRAM is suitable as an application with few writing operations and frequent read operations. Therefore, the performance of the sensing amplifier schemes is the most critical evaluation for RRAM.

**Figure 7:**
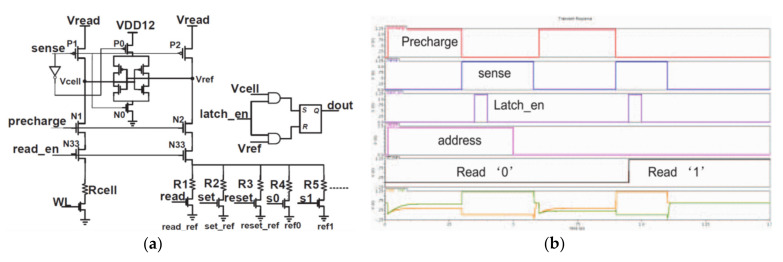
(**a**) Latch type sensitive amplifier; (**b**) operation waveform [80].

Figure 7a shows the traditional latch-sensitive amplifier [80]. Firstly, a voltage difference is established between Vcell and Vref, that is, a sensing margin. In the sensing phase, the sense amplifier is controlled by the PG and NG signals. The voltages of Vref and Vcell are driven to VDD or GND, respectively. The voltage-type sensitive amplifier includes a programmable reference generator, which can be connected to different reference resistors under different enable signals to satisfy the write-verify-write mode. Moreover, the sensing margin can also be adjusted according to different operations. However, the speed of this conventional amplifier is slow, and the sensing margin is small. It cannot work at low voltage and adapt to the small R-ratio.

As the supply voltage and R-ratio becomes small, the current difference between the LRS cell and the HRS cell will decrease. Therefore, it is necessary to extend the settling time of the BL to increase the voltage sensing margin, which may slow down the operation of reading. To improve the sensing margin, reduce settling time, and increase speed, Meng-Fan Chang et al., proposed a swing-sample-and couple voltage-mode sense amplifier (SSC-VSA) [81].

**Figure 8:**
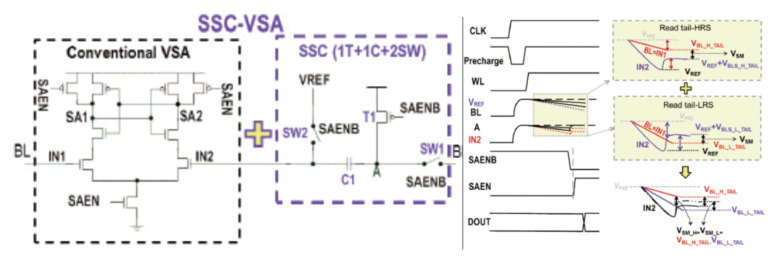
The structure and operation waveform of SSC-VSA [81].

This adds an extra PMOS transistor (T1), two switches (SW1, SW2), and a capacitor (C1) to the traditional voltage-type sensitive amplifier shown in Figure 8. It makes $V_{BLS}=VDD-V_{BL}$, $V_{REF}=VDD-(V_{BLS-H}+V_{BLS-L)}$. These tail-cells are written by the operation of programming verification.

In the first stage, V_BLS_ is sampled, and V_BL_ is applied to node IN1. The node A of C1 and IN2 is biased at VREF. In the second stage, the connection between node A and BL is cut off by switching off SW1. At the same time, T1 is turned on to raise the voltage of node A to VDD. Because of the capacitor, the voltage at point B is raised to V_REF_ + VDD-V_BL_ = V_REF_ + V_BLS_. In the third stage, the sensitive amplifier starts to work. It can be found that at this time $\Delta V_{SM}=V_{REF}+V_{BLS}-V_{BL}=2\times V_{BLS}-(V_{BLS-H}+V_{BLS-L})$, which becomes twice the traditional sensing margin $V_{BLS}=\frac{1}{2}(V_{BLS-H}+V_{BLS-L})$, showing that the sensing margin and reading speed have been improved. 

**Figure 9:**
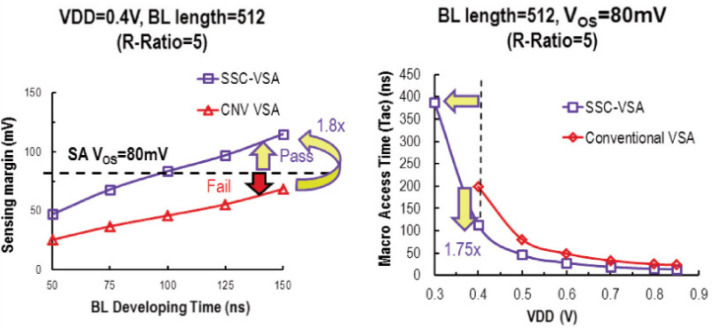
The performance of SSC-VSA (**a**) the sensing margin of SSC-VSA and CNV VSA at different BL developing time (**b**) the macro access time of SSC-VSA and conventional VSA at different VDD [81].

Figure 9a shows that, at VDD = 0.4 V, BL-length = 512, and R-ratio = 5, the sensing margin of SSC-VSA is larger than conventional VSA and the BL developing time is shorter. Moreover, SSC-VSA can achieve a 1.8 to 2× larger sensing margin than CNV-VSA at the same condition.

Figure 9b shows that, at BL length = 512 and VOS = 80 mV, SSC-VSA can achieve 100 mV lower VDD_MIN_ and 1.7 faster access time than CD-VSA for reading tail bits with R-ratio = 5. The measured T_AC_ at VDD = 0.85 V and 0.27 V are 6.8 ns and 404.4 ns, respectively.

**Figure 10:**
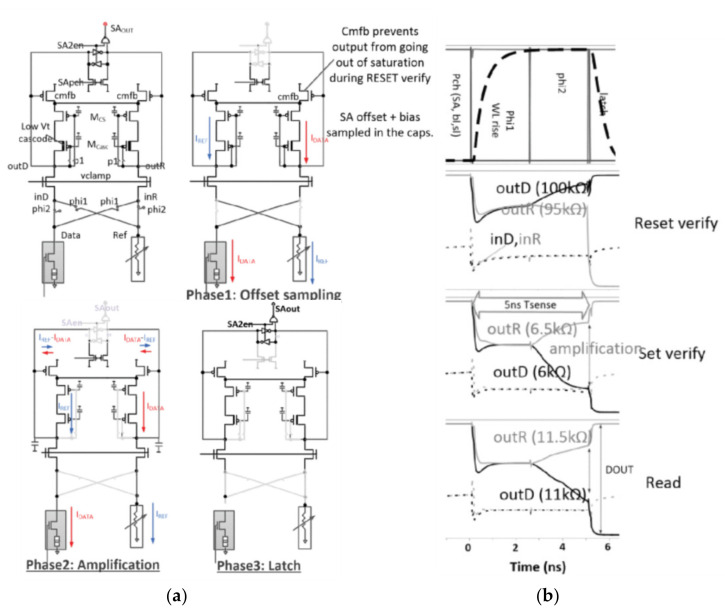
(**a**) OC-CSA in different phases; (**b**) example read, set, and reset-verify simulations [82].

The SSC-VSA increases the sensing margin, but the offset current still affects the sensing amplifier schemes. Therefore, Pulkit Jain et al., proposed an offset-canceling current sense amplifier (OC-CSA), which uses a cross-sampling technique to eliminate the influence on the offset on the data path of the RRAM cell shown in Figure 10 [82].

In phase-1, phi1 is on, Idata flows through the path on the right, and Iref flows through the path on the left. Due to the diode connection mode, the current bias voltage and the offset voltages caused by the path are all sampled to the capacitors Mcs and Mcasc.

In phase-2, phi1 is turned off and phi2 is turned on. Idata flows through the left path, and Iref flows through the right path, so the current flowing through the second-order latch sampling point is Idata-Iref and Iref-Idata. The difference is 2 × (Idata-Iref), and it also reduces the effect of the offset voltage.

In phase-3, the second-stage sensitive amplifier starts to work, and the sampling point is pulled to VDD or GND.

The common-mode feedback module can effectively ensure that the first-stage amplifier can also be in the correct working state when the current is small, and using the cascade diode connection form can enhance the suppression of VDD power noise. It can be realized at 0.7 V and the sensing time is 5 ns.

**Figure 11:**
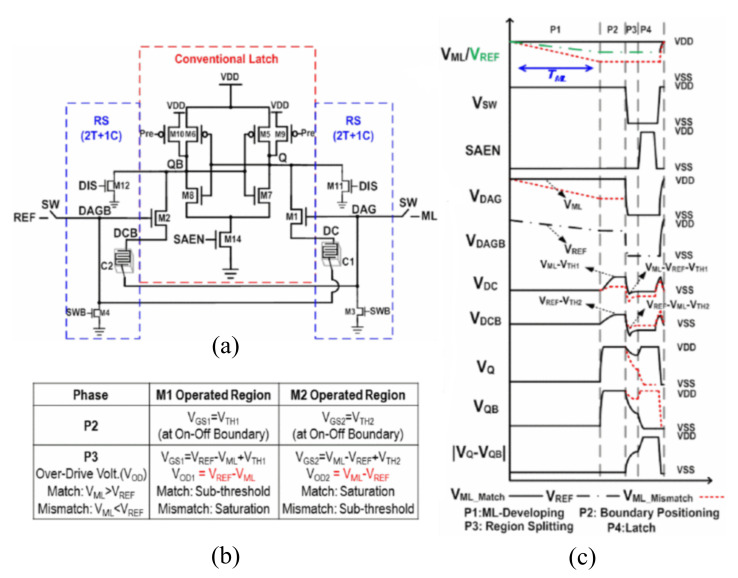
(**a**) The structure of RS-SA; (**b**) the state of sampling transistor; (**c**) the simulation waveform [83].

Although the offset current caused by the parasitic capacitor and resistance of the path is eliminated, the two sampling transistors may cause mismatch and drift of the threshold voltage due to process fluctuations. Therefore, Chien-Chen Lin et al., proposed the region-splitter sense amplifier (RS-SA) shown in Figure 11 [83]. The use of capacitors C1 and C2 eliminates the mismatched threshold voltage and also achieves the enlarged difference between the two sides of the voltage.

In the first stage, M3 and M4 are turned off, SW is turned on, and the voltages of DAGB and DAG are V_REF_ and V_ML_, respectively. At the same time, M11 and M12 are turned on, and the voltages at Q and QB are pulled down to zero. In the second stage, M9 and M10 are turned on to pull Q and QB up to the VDD. Moreover, due to the limitations of M1 and M2, the voltages of DCB and DC are V_REF_-V_TH2_, V_ML_-V_TH1_. In the third stage, M3, M4 are turned on, and the voltages of DAG and DAGB are pulled down to zero. Through the boost of capacitors C1 and C2, the voltages at DCB and DC are V_REF_-V_TH2_-V_ML_, V_ML_-V_TH1_-V_REF_, respectively, so the over-driving voltages of M1, M2 are V_REF_-V_ML_, V_ML_-V_REF_, respectively. It is not related to the threshold voltage anymore. Therefore, the mismatch caused by the threshold voltage is eliminated. In addition, the states of M1 and M2 are the saturated region or sub-threshold region. Therefore, the current passing through will have a big difference. Within a certain period, the voltages at Q and QB will have a large sensing window.

**Figure 12:**
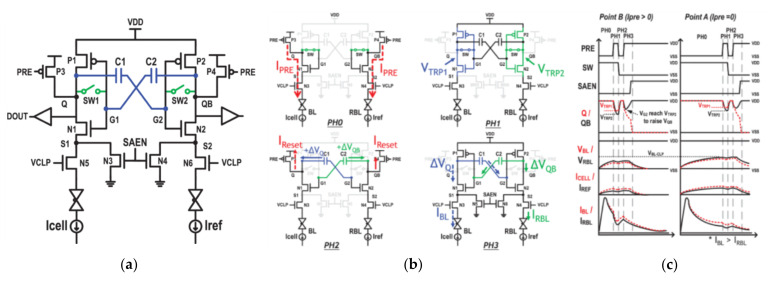
(**a**) The scheme of DTPMS; (**b**) the operation of DTPMS; (**c**) the simulation of DTMPS [84].

The above methods can eliminate the mismatch on the paths and sampling transistors, but the impact on the trip-point voltage of the latch has not been eliminated. Therefore, Chieh-Pu Lo et al., proposed dynamic trip-point-mismatch sampling (DTPMS) CSA shown in Figure 12 [84]. It eliminates the offset of trip point voltage through capacitors C1 and C2.

First, SW is turned off, and BL and BLR are charged to the clamping voltage through P3 and P4; then, P3 and P4 are turned off, and SW is still closed at this time. At this time, the gate voltages of P1 and N1 are the trip point voltage (V_TRP1_), and V_TRP2_ is generated in the same way. As a result, $\Delta V=V_{TRP1}-V_{TRP2}$ is stored on C1 and −ΔV is stored on capacitor C2. In the next stage, to ensure that the voltages at points Q and QB are the same, turn on P3 and P4 again, and turn off SW at the same time. It raises the voltages at Q and QB to VDD, while the voltage at G1 is pulled up to $V_{VTP1}+(VDD-V_{VTP2})$, and the voltage of G2 is pulled up to $V_{VTP2}+(VDD-V_{VTP1})$, due to the capacitor. The next stage is sensing and outputting the results.

Assuming that the read cell is LRS, therefore ILRS > IREF. The V_TRP1_ is higher than V_TRP2_ (worst case for reading LRS). If VQ changes, VDD-V_TRP1_, the voltage at point Q is 2 V_TRP1_-V_TRP2_ > V_TRP1_, but the voltage at point QB changes to V_TRP2_ and reaches the flip point. Thus, despite the mismatch against reading LRS (V_TRP1_ > V_TRP2_), the P2-N2 pair are activated ahead of the P1-N1 pair and still generate correct sensing operation. A fabricated 65 nm 2 Mb ReRAM macro achieved T_CD_ = 2.6 ns at VDD = 1 V.

**Figure 13:**
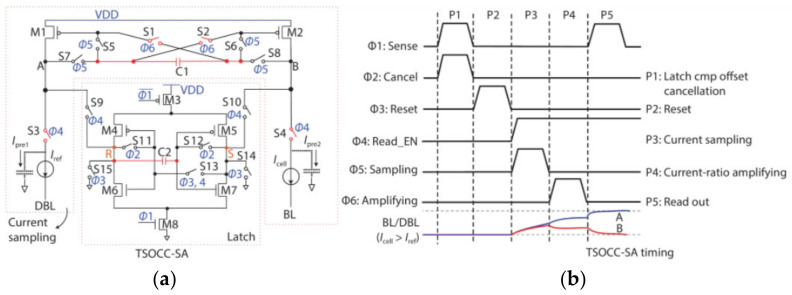
(**a**) The scheme of TSOCC-SA; (**b**) the simulation of TSOCC-SA [77].

The capacitor has a huge effect on eliminating mismatch and removing the offset voltage. However, the capacitor will take up a lot of areas. Therefore, Qiao wang et al., propose the two-stage offset-cancelled current sense amplifier (TSOCC-SA) shown in Figure 13 [77].

It eliminates not only the mismatch of M1, M2 but also the mismatch of latch trip voltage with only two capacitors. It uses capacitor C1, switches S1 and S2 to eliminate the mismatch of M1 and M2, and switches S3 and S4 to expand the sensing margin. Use capacitor C2 to eliminate the mismatch of latch trip voltage.

In the P1 period, the two inverters’ input and output are connected, respectively. Therefore, the capacitor C2 samples the difference between the trip voltages of the two inverters. In the P2 period, “0” voltage is reset to the outputs of the two inverters and the inputs of the left inverter. By the boost of C2, the input of the right inverter becomes “VTR—VTL”. In the P3 period, two diode-connected transistors (M1 and M2) charge (Ipre1 and Ipre2) the A and B nodes. After a sufficient precharge time, the currents of M1 and M2 (IM1 and IM2) decrease to near Iref and Icell. Finally, the gate voltages (VG1 and VG2) of M1 and M2 are stored at the left and right ends of capacitor C1. In the P4 period, the two switches (S1 and S2) are turned on, and the four switches (S5–S8) are turned off. The A (B) node has a current path to GND through S3 (S4), resulting in strong positive feedback. In the P5 period, the latched comparator starts to work and outputs the data.

In the TSOCC-SA, the M1 (M2) charges the A (B) node with the sampled current Icell (Iref), while the S3 (S4) discharges the A (B) node with the current Iref (Icell). Thus, the current difference between the node A and node B nodes is 2|Iref-Icell|, which is twice the sensing margin of conventional CSA.

**Figure 14:**
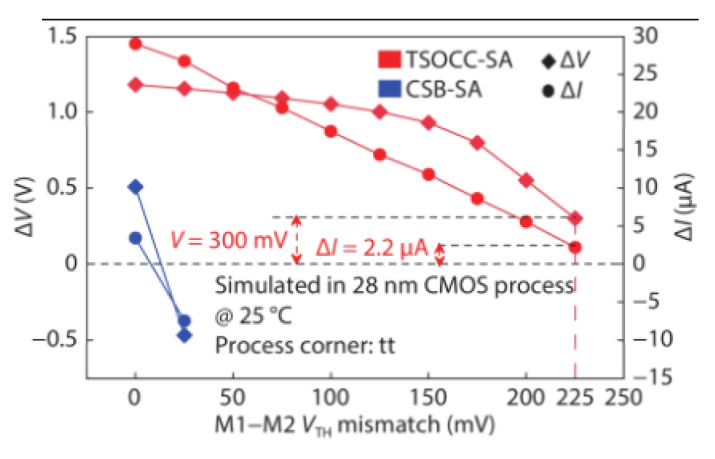
Simulated ΔV and ΔI vs. VTH mismatch between transistors M1 and M2 [77].

Figure 14 shows that when the device mismatch reaches 225 mV, the sensing margin is still enough, and the sense amplifier is valid. The performance in offset-tolerance of the TSOCC-SA is better than that of the CSB-SA in [85].

The mismatch between M4 and M5, M6 and M7 in the latched comparator will lead to the invalidation of SA. Hence, it is extremely necessary to introduce the cross-coupling capacitor C2 in the latched comparator to eliminate the mismatches and to improve the accuracy of SA.

Thus, TSOCC-SA uses only two capacitors to eliminate the mismatch of SA and data path. However, the operation of the scheme is complex, and the peripheral scheme which generates the control signal is somewhat difficult to design.

In order to further reduce power consumption, it is necessary to eliminate the offset current and accommodate a smaller input margin. Taehui Na proposed offset-canceling single-ended sensing schemes (OCSE-SS) with one-BL precharge architecture (1 BLPA) [86].

Compared with the traditional structure, the energy required to read the operation is only a quarter of the original. The traditional readout scheme requires two sensing paths, leading to high readout energy. Particularly in deep-sub micrometer technology nodes, the mismatch of the transistor caused by process fluctuations will result in a relatively large offset voltage.

**Figure 15:**
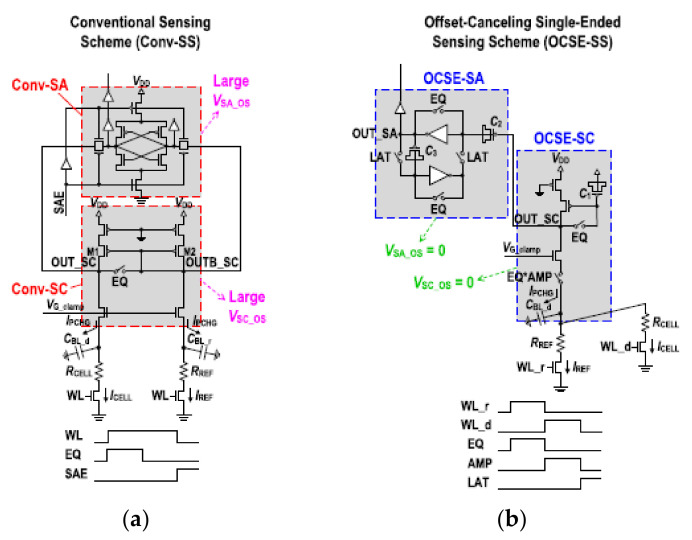
(**a**) Two-BL precharge architecture (2 BLPA) differential-sensing structure with no offset voltage cancellation; (**b**) one-BL precharge architecture (1 BLPA) single-ended sensing structure offset voltage cancellation [86].

Figure 15 shows the scheme of the OCSE-SS with 1 BLPA consisting of an offset-canceling single-ended SC (OCSE-SC) and an offset-canceling single-ended SA (OCSE-SA). The effect of mismatch is eliminated due to sensing schemes adopting a single-ended mode. In addition, capacitors C2 and C3 are used to eliminate the offset of SA. Therefore, the effect of offset in this SA is completely eliminated.

In the first stage, WL_r is turned on and Iref is sampled. Vref is stored in capacitor C1, and EQ is turned off, V_TRP1_ and V_TRP2_ are established at both ends of C3. Voffset is stored in capacitors C2 and C3, which will eliminate the impact of the offset. After this time, WL_d is on and Icell will flow in and compare with the Iref stored on C1. At this time, the margin is small, but it will be amplified by the two-stage inverter and stored in the corresponding node, then the LAT will be turned off and SA will output the data.

**Figure 16:**
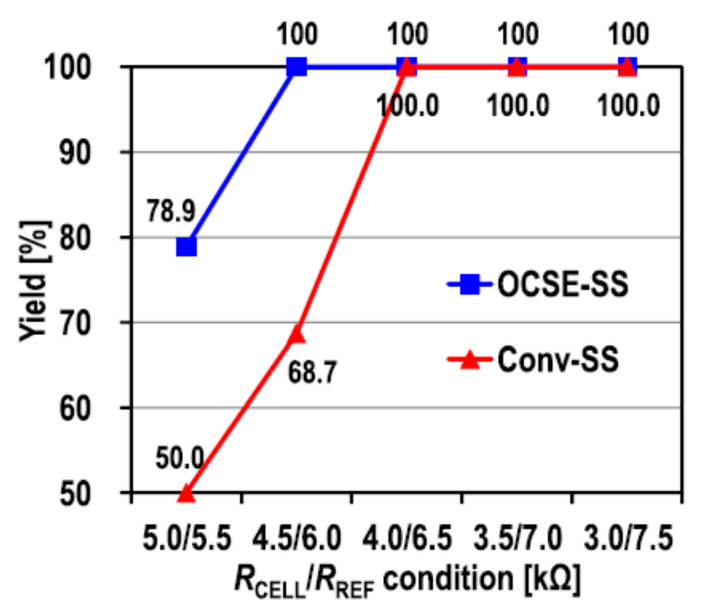
Measured yield versus RCELL/RREF of the Conv-SS and the OCSE-SS at VDD = 0.9 V, VG_clamp = 0.8 V, and t_SEN_ = 20 ns [86].

At the 65 nm process node, the read energy/bit is only 241 fJ, the VDD is 0.9 V, and the minimum R difference resolution is 1.5 KΩ shown in Figure 16. However, the robustness of this scheme is a little poor and it is sensitive to the noise in the scheme because of the high resolution.

As the supply voltage decreases, the effective voltage applied to the cell becomes smaller. One of the solutions is to enhance the sensing ability to overcome the challenge of a small read-out window, as mentioned above. Another solution is reducing the voltage headroom of sensing schemes to increase the effective voltage applied to the cell. Based on this idea, Meng-Fan Chang et al., proposed the body-drain-driven (BDD) read scheme [87].

**Figure 17:**
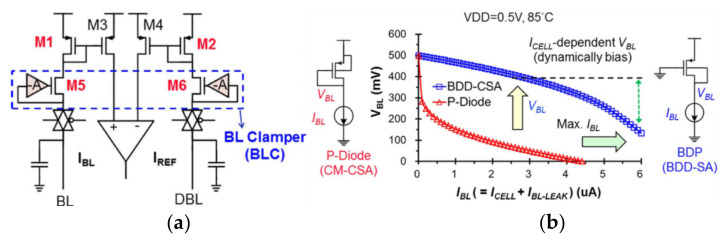
(**a**) The traditional sensing scheme; (**b**) VBL of BDD-SA and CM-CSA across various IBL [87].

**Figure 18:**
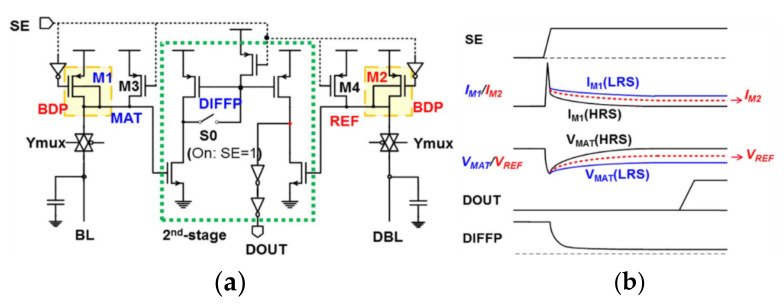
(**a**) The schemes of BDD-CSA; (**b**) the operation of BDD-CSA [87].

Figure 17a shows the traditional sensing scheme. The voltage on BL is that VDD subtracts the voltage consumed by M1 and M5. The sensing scheme uses the body-drain-driven mode to replace the diode connection mode, which increases voltage headroom on BL and the Figure 17b shows that at I_BL_ = 3 uA, BDD-CSA can save 300 mV voltage headroom than P-Diode. Moreover, the BDD sensing scheme removes the clamp transistor M5/M6 by using unipolar RRAM cells to extend the upper limit of VBL. Figure 18a shows the scheme of BDD-CSA. It operates in the following three phases.

In phase-1, S0 and YUMX are turned on. The node voltages at MAT and REF are pulled down by the heavy load on BL/DBL. Then, the transistors M1 and M2 begin to precharge the BL and DBL, respectively.

In phase-2, after sufficient precharge time, V_MAT_ and V_REF_ come to the target level. At the end of phase-2, VMAT and VREF are different, due to the difference between IM1 and IM2.

In phase-3, the amplifier detects the voltage difference between V_MAT_ and V_REF_ and then generates a large signal output.

Moreover, the parasitic diodes located on the source side of M1 and M2 expand the functionality across a wide range of supply voltage.

**Figure 19:**
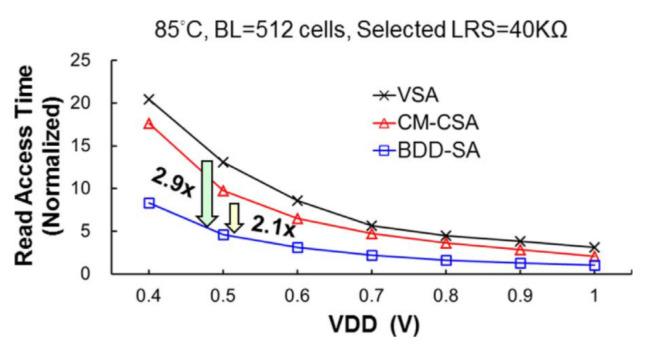
The normalized read access time of a 65 nm 4 Mb macro using various sense amplifiers [87].

Figure 19 shows that the read speed of BDD-SA is 2.9× faster than voltage-mode SA and 2.1× faster than traditional current-mode SA at VDD = 0.5 V. At VDD = 0.5 V, the read access time is 45 ns. It can achieve read operation at VDD = 0.32 V. However, this scheme does not cancel the effect of the offset current and adapts to the small R-ratio.

### 3.3. BL-Enhancing Schemes

In addition to improving the recognition accuracy, the sensing speed of the sensing amplifier, reducing its power consumption and area, BL-enhancing schemes, which can stabilize the voltage and speed up the charging speed on the BL, are important for sensing schemes.

**Figure 20:**
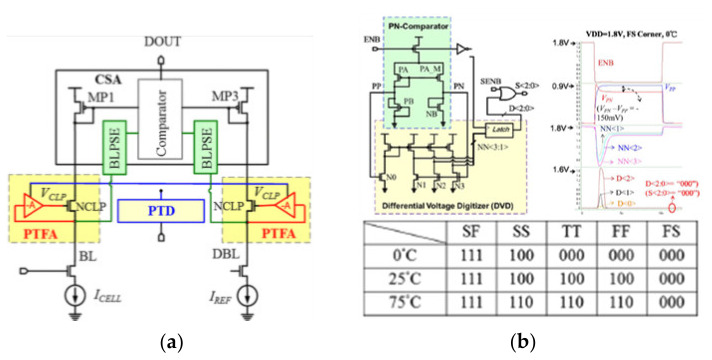
(**a**) The structure of PTADB; (**b**) the structure and simulation of PTD. The table is the coding of different process corners and temperatures [76].

Meng-Fan Chang et al., proposed the process temperature-aware dynamic BL-bias scheme (PTADB), in order to achieve smaller VBL fluctuations, prevent read disturb, and faster BL charge time. It contains a process temperature detector (PTD), a process temperature-compensated feedback amplifier (PTFA), and a BL precharge speed enhancement (BLPSE) scheme shown in Figure 20a.

The PTD scheme includes a differential digital counter and a PMOS-NMOS(P-N) intensity comparator. The P-N comparator includes a current mirror, diode connection mode of PMOS and NMOS. The current mirror copies the current flowing through the PMOS to the NMOS to make a different voltage at node PN. Under different process corners and temperatures, the driving strengths of PMOS and NMOS are different. Then the voltage of node PN is delivered to the DVD (differential voltage digitizer). According to the value of the PN node voltage, the signal S < 2:0> is generated. The signal S is related to the process and temperature. The table in Figure 20b shows the coding of different process angle and the temperature. However, the coding of different process corners and temperatures is a little simple.

**Figure 21:**
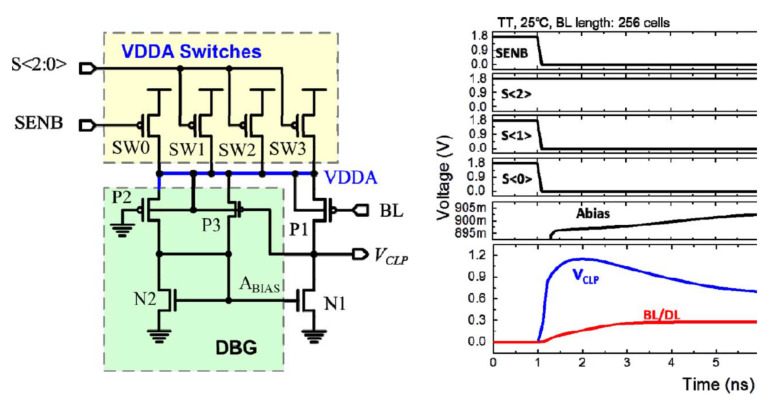
The structure and simulation of PTFA [76].

The (process temperature compensated feedback amplifier) PTFA scheme is used to dynamically adjust the gate voltage of the clamp transistor NLP according to the process corner and temperature information measured by the PTD scheme shown in Figure 21. The VDDA voltage in the PTFA scheme is modulated by the signal S. PTFA contains a dynamic bias generator (DBG) scheme, which can dynamically provide the gate voltage of N1 so that VCLP is at a variable value instead of a fixed value. In the beginning, the VCLP voltage is large, which can increase the charging speed of BL. The PTD and PTFA schemes are used together to reduce voltage fluctuations on BL caused by process fluctuations and relieve read disturb. It can reduce the variation by 45% and 56%, compared with the conventional (CNV) dynamic BL bias scheme and our PTFA without PTD, respectively.

**Figure 22:**
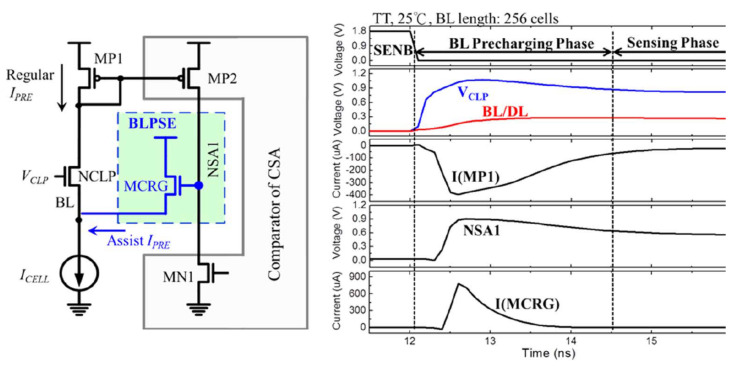
The structure and simulation of BLPSE [76].

The BLPSE (BL precharge speed enhancement) scheme is used to reduce the charge time of the BL, without affecting the sensing margin, shown in Figure 22. In the initial stage of charge, a large current is generated on BL (to charge parasitic capacitance), and it is copied to MP2, then the voltage of NSA1 is raised, the MCRG transistor is turned on, and the charging speed of BL accelerates. After BL reaches the target value, the current decreases. The voltage of node NSA1 drops. The MCRG transistor is turned off.

**Figure 23:**
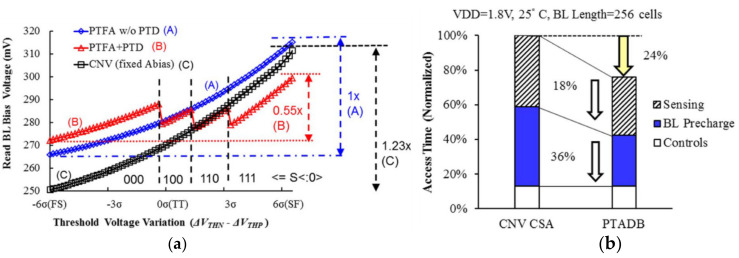
(**a**) BL bias voltage across various process corners; (**b**) read access time of CNV-CSA and PTADB [76].

Figure 23a shows that the variation in voltage of BL is reduced by 45% and 56% compared with the conventional scheme and PTFA without PTD.

Figure 23b shows that it achieves a 36% and 18% reduction in BL precharge time and sensing time compared to CNV-CSA, respectively, by using PTFA and BLPSE schemes. Moreover, the read access time of PTADB is reduced by 24%.

In addition to process and temperature fluctuations, power supply voltage fluctuations and load changes can also cause apparent ripples in the voltage on the BL, which may affect the stability of RRAM. Therefore, optimizing the drive power is essential to achieve a stable BL voltage. Chung-Cheng Chou et al., proposed the low-ripple charge pump scheme (LR-CP) [78].

**Figure 24:**
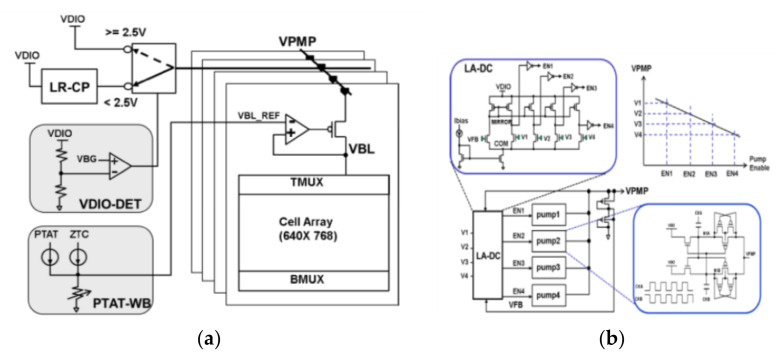
(**a**) Macro architecture; (**b**) the structure of the low-ripple charge pump [78].

The RRAM Cell Array shares a local LDO. The power supply of LDO can be VDIO or a low-ripple charge pump (LR-CP) controlled by VDIO Detection (VDIO-DET). When the voltage of VDIO is less than 2.5 V, the LR-CP works. Because the traditional switched capacitor charge pump is difficult to obtain a low output ripple, a load sensing detection circuit (LA-DC) in Figure 24b is proposed to solve this problem, which can adaptively deploy the required pump according to the current load. Under a light load condition, one pump is activated to keep the VPMP to its maximum (V1). When the current load exceeds the driving capacity of one pump, the VPMP will drop and trigger more pumps in turn.

**Figure 25:**
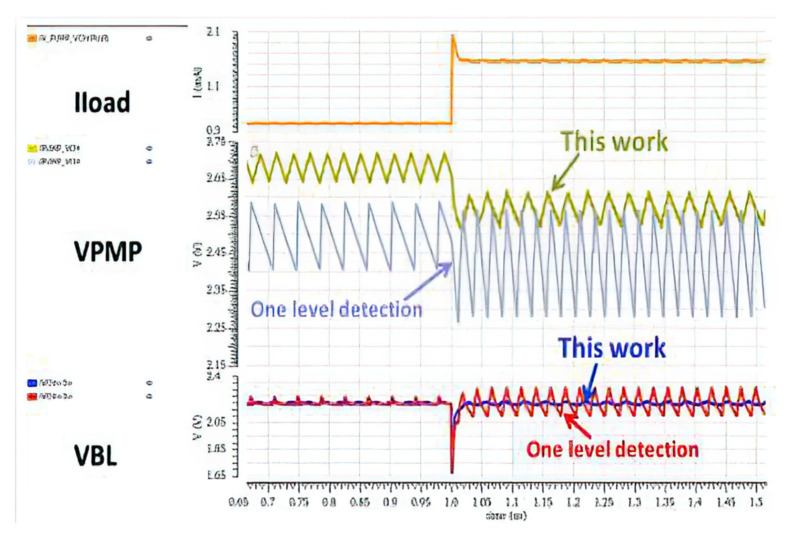
Transient comparison between with and without LA-DC [78].

Figure 25 shows the transient performance of conventional one-level detection compared to the LA-DC. Moreover, it realizes a consistent write performance at an operating voltage range of 1.62 V~3.63 V and reduces the read disturb with only a capacitor is 20 pF. However, it is only suitable for a high working voltage at more than 1.6 V and cannot adapt to the low or ultra-low voltage.

### 3.4. The Summary of Sensing Schemes

Table 2 is the summary of the sensing schemes. It can be a design guide. The designers can choose different schemes to meet the requirement.

## 4. Summary and Outlook

In the past few years, RRAM has been applied to the more and more advanced technology nodes—from 180 nm to 14 nm. Moreover, its application range is wide, including high-speed memory, low-power embedded memory, and brain-inspired computing.

This paper introduces several novel schemes and level techniques to deal with these difficulties in optimizing the reading performance. As for reference schemes, HRRS, 4T3R reference, and MCDC are introduced to make a tight current, achieve temperature tacking, and adapt to tail-bit issues, respectively. Then, this paper summarizes some sensing amplifier schemes; they can be used to enlarge the sensing margin, cancel the offset current, work at ultra-low voltage, and reduce the area and power consumption. Last, a few BL-enhancing schemes are present to lower the effect of temperature-process variation and the ripple on BL. Moreover, this paper also explains the weakness of each method.

In the future, the development and perspectives for the design of sensing schemes should include the following:

(1) Reference schemes should track the RRAM cell in not only temperature but also time variation, and deal with the tail-bits issue occurring in reference schemes.
(2) Sensing amplifier schemes should achieve high resolution, strong robustness, high speed, and work at ultra-low voltage.
(3) BL-enhancing schemes should stabilize the BL voltage, ensure enough efficient BL voltage, and work at ultra-low voltage.

## Figures and Tables

**Table 1:** Published articles on RRAM’s periphery schemes since 2012. The first column lists the first author, publishing year, and journal abbreviation, together with the first affiliation shown in the parenthesis.

Reference	Cell Structure/ Storge Size (Bite)/ Technology Node	Speed/Programming Condition	Optimization of Scheme
X.Y.Xue 2012 VLSI (Fudan)	1T1R/8 M/130 nm	TAC = 21 ns@VDD = 1.2 V	self-adaptive write mode (SAWM) self-adaptive read mode (SARM)
Meng-Fan Chang 2013 JSSC (NTHU)	1T1R/4 M/180 nm	TAC = 7.2 ns@VDD = 1.8 V	parallel-series reference cell (PSRC) process temperature-aware dynamic BL-bias (PTADB) schemes
Sung Hyun Jo 2014 IEDM (Crossbar)	1S1R/4 M/100 nm	NA	NA
Meng-Fan Chang 2014 ISSCC (NTHU)	1T1R/1 M/28 nm	TAC = 6.8 ns@VDD = 0.85 V TAC = 404 ns@VDD = 0.27 V	swing-sample-and couple voltage-mode sense amplifier (SSCVSA) self-boost-write-termination(SBWT)
Tz-yi Liu 2014 JSSC (Sandick)	1D1R/32 G/24 nm	NA	A dynamic charge pump control scheme Smart Read approach Write scheme and leakage compensation
Wei-Hao Chen 2017 IEDM (NTHU)	1T1R/16 M/150 nm	NA	self-write termination scheme (SAWM)
Chung-Cheng Chou 2018 ISSCC (TSMC)	1T1R/11 M/40 nm	TAC = 9 ns	a low voltage write-current-limiting scheme (LV-WCLS) SL-Precharge SA
Chieh-Pu Lo 2019 JSSC (NTHU)	1T1R/2 M/65 nm	TCD = 2.9 ns	dynamic trip-point-mismatch sampling (DTPMS) scheme a low dc current voltage-mode write termination (LDC-VWT)
Pulkit Jain 2019 ISSCC (Intel)	1T1R/NA/22nm	TAC < 5 ns@VDD = 0.7 V TAC < 10 ns@VDD = 0.5 V	pulse-width (PW) voltage-current write-verify-write (PVC-WVW) offset cancelling current sense amplifier(OC-CAS)
Chung-Cheng Chou 2020 VLSI (TSMC)	1T1R/13.5 M/22 nm	TAC = 6.5 ns@VDD = 0.7 V	Low-Ripple Charge Pump Scheme The Hybrid Self-Tracking Reference
Jianguo Yang 2020 VLSI (IMCAS)	1T2R/1.5 M/28 nm	TAC = 3.3 ns@VDD = 0.8 V	1T2R cell using PMOS selector hierarchical bitline and 3-state cell storage self-adaptive write with current limiter and sneaking current compensator reverse read with dummy ref.
Jianguo Yang 2021 ISSCC (IMCAS)	1T1R/1 M/14 nm	TAC = 9.5 ns@VDD = 0.8 V TAC = 21 ns@VDD = 0.4 V	Array Architecture Self-adaptive delayed termination (SADT) Multi-Cell Reference

**Table 2:** The summary of sensing schemes and the advantages and weaknesses of each scheme.

The Category of Sensing Schemes	The Acronym of Sensing Schemes	Advantages	Weaknesses
Reference Schemes	Traditional reference	Simple	The poor tracking ability
	PSRC	Large R-ratio	Cannot adopt to tail bit
	HRRS	Tight reference current	Cannot adopt to tail bit
	4T3R reference	Good temperature tracking ability	Cannot adopt to tail bit
	MCDC	Adopt to tail bit	Complex
Sensing Amplifier Schemes	Traditional SA	Simple	Low speed, offset current, small sensing margin, high working voltage
	SSC-VSA	Larger sensing margin	Offset current
	OC-CSA	Cancel the offset at data path	Offset current
	RS-SA	Cancel the offset at sampling transistors	Offset current
	DTPMS-CSA	Cancel the offset at latch	Large area
	TSOCC-SA	Small area	Complex
	OCSE-SS	Low power consumption	A little poor robustness
	BDD-CSA	Low working voltage	Large R-ratio offset current
BL Enhancing Schemes	PTADB	Process temperature-aware	Simple coding
	LR-CP	Low ripple at BL	High working voltage

## Data Availability

The data that support the findings of this study are available from the corresponding author upon request.

## References

[1] Jefremow M., Kern T., Backhausen U., Peters C., Parzinger C., Roll C., Kassenetter S., Thierold S., Schmitt-Landsiedel D. Bitline-capacitance-cancelation sensing scheme with 11 ns read latency and maximum read throughput of 2.9 GB/s in 65 nm embedded flash for automotive. Proceedings of the 2012 IEEE International Solid-State Circuits Conference.

[2] Chang M.-F., Shen S.-J. (2009). A process variation tolerant embedded split-gate flash memory using pre-stable current sensing scheme. IEEE J. Solid State Circuits.

[3] Kanda A., Kurafuji T., Takeda K., Ogawa T., Taito Y., Yoshihara K., Nakano M., Ito T., Kondo H., Kono T. (2019). A 24-MB Embedded Flash System Based on 28-nm SG-MONOS Featuring 240-MHz Read Operations and Robust Over-the-Air Software Update for Automotive Applications. IEEE Solid State Circuits Lett..

[4] Chang M., Lin Y.-F., Liu Y.-C., Wu J.-J., Shen S.-J., Tsai W.-C., Chih Y.-D. (2015). An Asymmetric-Voltage-Biased Current-Mode Sensing Scheme for Fast-Read Embedded Flash Macros. IEEE J. Solid State Circuits.

[5] Halupka D., Huda S., Song W., Sheikholeslami A., Tsunoda K., Yoshida C., Aoki M. Negative-resistance read and write schemes for STT-MRAM in 0.13 µm CMOS. Proceedings of the 2010 IEEE International Solid-State Circuits Conference (ISSCC).

[6] Jefremow M., Kern T., Allers W., Peters C., Otterstedt J., Bahlous O., Hofmann K., Allinger R., Kassenetter S., Schmitt-Landsiedel D. Time-differential sense amplifier for sub-80 mV bitline voltage embedded STT-MRAM in 40 nm CMOS. Proceedings of the 2013 IEEE International Solid-State Circuits Conference Digest of Technical Papers.

[7] Noguchi H., Ikegami K., Takaya S., Arima E., Kushida K., Kawasumi A., Hara H., Abe K., Shimomura N., Ito J. 7.2 4 Mb STT-MRAM-based cache with memory-access-aware power optimization and write-verify-write/read-modify-write scheme. Proceedings of the 2016 IEEE International Solid-State Circuits Conference (ISSCC).

[8] Yang Z.-H., Li K.-X., Chiang Y.-N., Lin W.-Y., Lin H.-T., Chang M.-F. A 28 nm 32 Kb embedded 2T2MTJ STT-MRAM macro with 1.3 ns read-access time for fast and reliable read applications. Proceedings of the 2018 IEEE International Solid-State Circuits Conference-(ISSCC).

[9] Kim C., Kwon K., Park C., Jang S., Choi J. 7.4 A covalent-bonded cross-coupled current-mode sense amplifier for STT-MRAM with 1T1MTJ common source-line structure array. Proceedings of the 2015 IEEE International Solid-State Circuits Conference (ISSCC) Digest of Technical Papers.

[10] Ogiwara R., Takashima D., Doumae S., Shiratake S., Takizawa R., Shiga H. (2015). Highly Reliable Reference Bitline Bias Designs for 64 Mb and 128 Mb Chain FeRAMs. IEEE J. Solid State Circuits.

[11] Khwa W.-S., Chang M.-F., Wu J.-Y., Lee M.-H., Su T.-H., Yang K.-H., Chen T.-F., Wang T.-Y., Li H.-P., Brightsky M. (2016). A resistance-drift compensation scheme to reduce MLC PCM raw BER by over 100× for storage-class memory applications. IEEE Int. Solid State Circuits.

[12] De Sandre G., Bettinl L., Pirola A., Marmonier L., Pasotti M., Borghi M., Mattavelli P., Zuliani P., Scotti L., Mastracchio G. A 90 nm 4 Mb embedded phase-change memory with 1.2 V 12 ns read access time and 1 MB/s write throughput. Proceedings of the 2010 IEEE International Solid-State Circuits Conference (ISSCC).

[13] Chang M.-F. A 0.5 V 4 Mb logic-process compatible embedded resistive RAM (ReRAM) in 65 nm CMOS using low-voltage current-mode sensing scheme with 45 ns random read time. Proceedings of the IEEE International Solid-State Circuits Conference (ISSCC) Digest of Technical Papers.

[14] Kawahara A. (2012). An 8 Mb multi-layered cross-point ReRAM macro with 443 MB/s write throughput. IEEE J. Solid State Circuits.

[15] Sheu S.-S. A 4 Mb embedded SLC resistive-RAM macro with 7.2 ns read-write random-access time and 160 ns MLC-access capability. Proceedings of the 2011 IEEE International Solid-State Circuits Conference.

[16] Govoreanu B., Kar G.S. 10 × 10 nm^2^ Hf/HfOx crossbar resistive RAM with excellent performance, reliability and low-energy operation. Proceedings of the 2011 International Electron Devices Meeting.

[17] Redolfi A., Goux L. A novel CBRAM integration using subtractive dry-etching process of Cu enabling high-performance memory scaling down to 10 nm node. Proceedings of the 2015 Symposium on VLSI Technology (VLSI Technology).

[18] Ho C.H., Hsu C.-L., Chen C.-C., Liu J.-T., Wu C.-S., Huang C.-C., Hu C., Yang F.-L. 9 nm half-pitch functional resistive memory cell with <1 µA programming current using thermally oxidized sub-stoichiometric WOx film. Proceedings of the 2010 International Electron Devices Meeting.

[19] Banno N., Tada T. A fast and low-voltage Cu complementary-atom-switch 1 Mb array with high-temperature retention. Proceedings of the 2014 Symposium on VLSI Technology (VLSI-Technology): Digest of Technical Papers.

[20] Govoreanu B., Di Piazza L. Advanced a-VMCO resistive switching memory through inner interface engineering with wide (>102) on/off window, tunable μA-range switching current and excellent variability. Proceedings of the 2016 IEEE Symposium on VLSI Technology.

[21] Tsunoda K., Kinoshita K., Noshiro H., Yamazaki Y., Iizuka T., Ito Y., Takahashi A., Okano A., Sato Y., Fukano T. Low Power and High Speed Switching of Ti-doped NiO ReRAM under the Unipolar Voltage Source of less than 3 V. Proceedings of the 2007 IEEE International Electron Devices Meeting.

[22] Goux L., Fantini A. Ultralow sub-500 nA operating current high-performance TiN\Al_2_O_3_\HfO_2_\Hf\TiN bipolar RRAM achieved through understanding-based stack-engineering. Proceedings of the 2012 Symposium on VLSI Technology (VLSIT).

[23] Govoreanu B., Redolfi A. Vacancy-modulated conductive oxide resistive RAM (VMCO-RRAM): An area-scalable switching current, self-compliant, highly nonlinear and wide on/off-window resistive switching cell. Proceedings of the 2013 IEEE International Electron Devices Meeting.

[24] Cheng C.-H., Tsai C.-Y., Chin A., Yeh F.S. High performance ultra-low energy RRAM with good retention and endurance. Proceedings of the 2010 International Electron Devices Meeting.

[25] Kim W. Forming-free nitrogen-doped AlOX RRAM with sub-μA programming current. Proceedings of the 2011 Symposium on VLSI Technology—Digest of Technical Papers.

[26] Xu X., Luo Q., Gong T., Lv H., Long S., Liu Q., Chung S.S., Li J., Liu M. Fully CMOS compatible 3D vertical RRAM with self-aligned self-selective cell enabling sub-5 nm scaling. Proceedings of the 2016 IEEE Symposium on VLSI Technology.

[27] Suri M., Bichler O. CBRAM devices as binary synapses for low-power stochastic neuromorphic systems: Auditory (Cochlea) and visual (Retina) cognitive processing applications. Proceedings of the 2012 International Electron Devices Meeting.

[28] Lee F.M., Lin Y.Y. A novel cross point one-resistor (0T1R) conductive bridge random access memory (CBRAM) with ultra low set/reset operation current. Proceedings of the 2012 Symposium on VLSI Technology (VLSIT).

[29] Vianello E., Thomas O. Resistive Memories for Ultra-Low-Power embedded computing design. Proceedings of the 2014 IEEE International Electron Devices Meeting.

[30] Luo Q., Xu X. Demonstration of 3D vertical RRAM with ultra-low-leakage, high-selectivity and self-compliance memory cells. Proceedings of the 2015 IEEE International Electron Devices Meeting (IEDM).

[31] Ho C.-H., Shen T.Y. Random soft error suppression by stoichiometric engineering: CMOS compatible and reliable 1 Mb HfO_2_-ReRAM with 2 extra masks for embedded IoT systems. Proceedings of the 2016 IEEE Symposium on VLSI Technology.

[32] Symanczyk R., Dittrich R. Conductive Bridging Memory Development from Single Cells to 2 Mbit Memory Arrays. Proceedings of the 2007 Non-Volatile Memory Technology Symposium.

[33] Goux L., Belmonte A. Retention disturb and variability improvements enabled by local chemical-potential tuning and controlled Hour-Glass filament shape in a novel W\WO_3_\Al_2_O_3_\Cu CBRAM. Proceedings of the 2016 IEEE Symposium on VLSI Technology.

[34] Chua L. (1971). Memristor-The missing circuit element. IEEE Trans. Circuit Theory.

[35] Strukov D.B., Snider G.S., Stewart D.R. (2008). The missing memristor found. Nature.

[36] Fackenthal R., Kitagawa M. 19.7 A 16 Gb ReRAM with 200 MB/s write and 1 GB/s read in 27 nm technology. Proceedings of the 2014 IEEE International Solid-State Circuits Conference Digest of Technical Papers (ISSCC).

[37] Sills S., Yasuda S. A copper ReRAM cell for Storage Class Memory applications. Proceedings of the 2014 Symposium on VLSI Technology (VLSI-Technology): Digest of Technical Papers.

[38] Zahurak J., Miyata K. Process integration of a 27 nm, 16 Gb Cu ReRAM. Proceedings of the 2014 IEEE International Electron Devices Meeting.

[39] Liu T.Y., Yan T.H. (2014). A 130.7-mm^2^ 2-Layer 32-Gb ReRAM Memory Device in 24-nm Technology. IEEE J. Solid State Circuits.

[40] Wu H. (2017). Resistive Random Access Memory for Future Information Processing System. Proc. IEEE.

[41] Waser R., Dittmann R. (2009). Redox-based resistive switching memories—Nanoionic mechanisms prospects and challenges. Adv. Mater..

[42] Kim K.M., Jeong D.S., Hwang C.S. (2011). Nanofilamentary resistive switching in binary oxide system: A review on the present status and outlook. Nanotechnology.

[43] Valov I., Waser R., Jameson J.R., Kozicki M.N. (2011). Electrochemical metallization memories—Fundamentals applications prospects. Nanotechnology.

[44] Jeong D.S. (2012). Emerging memories: Resistive switching mechanisms and current status. Rep. Prog. Phys..

[45] Xu X. First Demonstration of OxRRAM Integration on 14 nm FinFet Platform and Scaling Potential Analysis towards Sub-10 nm Node. Proceedings of the 2020 IEEE International Electron Devices Meeting (IEDM).

[46] Govoreanu B. Performance and reliability of Ultra-Thin HfO_2_-based RRAM (UTO-RRAM). Proceedings of the IEEE International Memory Workshop.

[47] Zhuo V.Y. (2013). Improved Switching Uniformity and Low-Voltage Operation in TaOx-Based RRAM Using Ge Reactive Layer. IEEE Electron. Device Lett..

[48] Wei Z. Highly reliable TaOx ReRAM and direct evidence of redox reaction mechanism. Proceedings of the 2008 IEEE International Electron Devices Meeting.

[49] Chang M. Challenges at circuit designs for resistive-type Nonvolatile memory and nonvolatile logics in mobile and cloud applications. Proceedings of the 2014 12th IEEE International Conference on Solid-State and Integrated Circuit Technology (ICSICT).

[50] Shang Y., Ohsawa T. Accurate Measurement of Sneak Current in ReRAM Crossbar Array with Data Storage Pattern Dependencies. Proceedings of the International Symposium on VLSI Technology Systems and Application (VLSI-TSA).

[51] Bae W., Yoon K.J., Hwang C.S., Jeong D.-K. (2016). A crossbar resistance switching memory readout scheme with sneak current cancellation based on a two-port current-mode sensing. Nanotechnology.

[52] Zidan M.A., Eltawil A.M., Kurdahi F., Fahmy H.A.H., Salama K.N. (2014). Memristor multiport readout: A closed-form solution for sneak paths. IEEE Trans. Nanotechnol..

[53] Ambrogio S. Data retention statistics and modelling in HfO_2_ resistive switching memories. Proceedings of the 2015 IEEE International Reliability Physics Symposium.

[54] Liang J., Wong H.P. (2010). Cross-point memory array without cell selectors—Device characteristics and data storage pattern dependencies. IEEE Trans. Electron. Devices.

[55] Lin Y., Yuan R. 3D vertical RRAM architecture and operation algorithms with effective IR-drop suppressing and anti-disturbance. Proceedings of the IEEE International Symposium on Circuits and Systems (ISCAS).

[56] Huang C., Xu N. (2021). Efficient and optimized methods for alleviating the impacts of IR-drop and fault in RRAM based neural computing systems. J. Electron. Devices Soc..

[57] Singh B., Davis L. An analysis of scale invariance in object detection–snip. Proceedings of 2018 IEEE/CVF Conference on Computer Vision and Pattern Recognition.

[58] Zhu Y., Zhao X. Insights and Optimizations on IR-drop Induced Sneak-Path for RRAM Crossbar-based Convolutions. Proceedings of the 2020 25th Asia and South Pacific Design Automation Conference (ASP-DAC).

[59] Xu C., Niu D., Muralimanohar N., Balasubramonian R., Zhang T. Overcoming the challenges of crossbar resistive memory architectures. Proceedings of the IEEE 21st International Symposium on High Performance Computer Architecture (HPCA).

[60] Hazra J., Liehr M., Beckmann K., Rafiq S., Cady N. Improving the Memory Window/Resistance Variability Trade-Off for 65 nm CMOS Integrated HfO_2_ Based Nanoscale RRAM Devices. Proceedings of the IEEE International Integrated Reliability Workshop (IIRW).

[61] Grossi A. (2015). Impact of Intercell and Intracell Variability on Forming and Switching Parameters in RRAM Arrays. IEEE Trans. Electron. Devices.

[62] Garbin D. Modeling of OxRAM variability from low to high resistance state using a stochastic trap assisted tunneling-based resistor network. Proceedings of the Joint International EUROSOI Workshop and International Conference on Ultimate Integration on Silicon.

[63] Chen A., Lin M. Variability of resistive switching memories and its impact on crossbar array performance. Proceedings of the International Reliability Physics Symposium.

[64] Park J., Jo M. (2010). Investigation of state stability of low-resistance state in resistive memory. IEEE Electron. Device Lett..

[65] Muraoka S., Ninomiya T. Comprehensive understanding of conductive filament characteristics and retention properties for highly reliable ReRAM. Proceedings of the 2013 Symposium on VLSI Technology.

[66] Pérez E., Grossi A. Temperature impact and programming algorithm for RRAM based memories. Proceedings of the IEEE MTT-S International Microwave Workshop Series on Advanced Materials and Processes for RF and THz Applications (IMWS-AMP).

[67] Puglisi F.M., Qafa A. (2015). Temperature Impact on the Reset Operation in HfO_2_ RRAM. IEEE Electron. Device Lett..

[68] Wu L. Nonlinear Weight Quantification for Mitigating Read Disturb Effect on Multilevel RRAM-Based Neural Network. Proceedings of the IEEE Electron Devices Technology & Manufacturing Conference (EDTM).

[69] Li X. Improvement of read disturb on TaOx-based RRAM cells with optimized pulse programming method. Proceedings of the IEEE International Conference on Integrated Circuits, Technologies and Applications (ICTA).

[70] Shim W., Luo Y., Seo J. Impact of Read Disturb on Multilevel RRAM based Inference Engine: Experiments and Model Prediction. Proceedings of the IEEE International Reliability Physics Symposium (IRPS).

[71] Su P.C., Jiang C.M., Wang C.W. Correlation between SET-state current level and read-disturb failure time in a resistive switching memory. Proceedings of the IEEE International Reliability Physics Symposium (IRPS).

[72] Na T., Song B., Kim J.P., Kang S.H., Jung S. (2017). Offset-Canceling Current-Sampling Sense Amplifier for Resistive Nonvolatile Memory in 65 nm CMOS. IEEE J. Solid State Circuits.

[73] Chang M. (2013). An Offset-Tolerant Fast-Random-Read Current-Sampling-Based Sense Amplifier for Small-Cell-Current Nonvolatile Memory. IEEE J. Solid State Circuits.

[74] Chen Y., Chin A. (2019). An Offset Readout Current Sensing Scheme for One-Resistor RRAM-Based Cross-Point Array. IEEE Electron. Device Lett..

[75] Luo Y., Chen W., Cheng M., Yin W. (2016). Electrothermal Characterization in 3-D Resistive Random Access Memory Arrays. IEEE Trans. Electron. Devices.

[76] Chang M. (2013). A High-Speed 7.2-ns Read-Write Random Access 4-Mb Embedded Resistive RAM (ReRAM) Macro Using Process-Variation-Tolerant Current-Mode Read Schemes. J. Solid State Circuits.

[77] Wang Q., Zhang D.L. (2021). Low-cost dual-stage offset-cancelled sense amplifier with hybrid read reference generator for improved read performance of RRAM at advanced technology nodes. J. Semicond..

[78] Chou C.C. A 22 nm 96KX144 RRAM Macro with a Self-Tracking Reference and a Low Ripple Charge Pump to Achieve a Configurable Read Window and a Wide Operating Voltage Range. Proceedings of the IEEE Symposium on VLSI Circuits.

[79] Yang J. A 14 nm-FinFET 1 Mb Embedded 1T1R RRAM with a 0.022 µm2 Cell Size Using Self-Adaptive Delayed Termination and Multi-Cell Reference. Proceedings of the IEEE International Solid-State Circuits Conference (ISSCC).

[80] Yang J. A 2 Mb ReRAM with two bits error correction codes circuit for high reliability application. Proceedings of the IEEE 10th International Conference on ASIC.

[81] Chang M. 19.4 embedded 1 Mb ReRAM in 28 nm CMOS with 0.27-to-1 V read using swing-sample-and-couple sense amplifier and self-boost-write-termination scheme. Proceedings of the IEEE International Solid-State Circuits Conference Digest of Technical Papers (ISSCC).

[82] Jain P. A 3.6 Mb 10.1 Mb/mm2 Embedded Non-Volatile ReRAM Macro in 22 nm FinFET Technology with Adaptive Forming/Set/Reset Schemes Yielding Down to 0.5 V with Sensing Time of 5 ns at 0.7 V. Proceedings of the IEEE International Solid-State Circuits Conference-(ISSCC).

[83] Lin C.C. A 256b-wordlength ReRAM-based TCAM with 1 ns search-time and 14× improvement in wordlength-energyefficiency-density product using 2.5T1R cell. Proceedings of the IEEE International Solid-State Circuits Conference (ISSCC).

[84] Lo C.P. (2019). A ReRAM Macro Using Dynamic Trip-Point-Mismatch Sampling Current-Mode Sense Amplifier and Low-DC Voltage-Mode Write-Termination Scheme against Resistance and Write-Delay Variation. IEEE J. Solid State Circuits.

[85] Chang M.F., Shen S.J. An offset-tolerant current-sampling-based sense amplifier for sub-100 nA-cell-current nonvolatile memory. Proceedings of the IEEE International Solid-State Circuits Conference.

[86] Na T., Song B. (2019). Offset-Canceling Single-Ended Sensing Scheme with One-Bit-Line Precharge Architecture for Resistive Nonvolatile Memory in 65-nm CMOS. IEEE Trans. Large Scale Integr. VLSI Syst..

[87] Chang M. (2013). A Low-Voltage Bulk-Drain-Driven Read Scheme for Sub-0.5 V 4 Mb 65 nm Logic-Process Compatible Embedded Resistive RAM (ReRAM) Macro. IEEE J. Solid State Circuits.

